# A new piroplasmid species infecting dogs: morphological and molecular characterization and pathogeny of *Babesia negevi* n. sp.

**DOI:** 10.1186/s13071-020-3995-5

**Published:** 2020-04-21

**Authors:** Gad Baneth, Yaarit Nachum-Biala, Adam Joseph Birkenheuer, Megan Elizabeth Schreeg, Hagar Prince, Monica Florin-Christensen, Leonhard Schnittger, Itamar Aroch

**Affiliations:** 1 Koret School of Veterinary Medicine, Hebrew University, P.O. Box 12, 76100 Rehovot, Israel; 2 North Carolina State University, Raleigh, NC USA; 3 Instituto de Patobiología Veterinaria, CICVyA, INTA-Castelar, 1686 Hurlingham, Argentina; 4 Consejo Nacional de Investigaciones Científicas y Técnicas (CONICET), Buenos Aires, Argentina

**Keywords:** *Babesia negevi* n. sp., Canine, Israel, *Ornithodoros tholozani*, *Borrelia persica*, *Babesia duncani*, *Babesia conradae*

## Abstract

**Introduction:**

Babesiosis is a protozoan tick-borne infection associated with anemia and life-threatening disease in humans, domestic and wildlife animals. Dogs are infected by at least six well-characterized *Babesia* spp. that cause clinical disease. Infection with a piroplasmid species was detected by light microscopy of stained blood smears from five sick dogs from Israel and prompted an investigation on the parasite’s identity.

**Methods:**

Genetic characterization of the piroplasmid was performed by PCR amplification of the *18S* rRNA and the cytochrome *c* oxidase subunit 1 (*cox*1) genes, DNA sequencing and phylogenetic analysis. Four of the dogs were co-infected with *Borrelia persica* (Dschunkowsky, 1913), a relapsing fever spirochete transmitted by the argasid tick *Ornithodoros tholozani* Laboulbène & Mégnin. Co-infection of dogs with *B. persica* raised the possibility of transmission by *O. tholozani* and therefore, a piroplasmid PCR survey of ticks from this species was performed.

**Results:**

The infected dogs presented with fever (4/5), anemia, thrombocytopenia (4/5) and icterus (3/5). Comparison of the *18S* rRNA and *cox*1 piroplasmid gene sequences revealed 99–100% identity between sequences amplified from different dogs and ticks. Phylogenetic trees demonstrated a previously undescribed species of *Babesia* belonging to the western group of *Babesia* (*sensu lato*) and closely related to the human pathogen *Babesia duncani* Conrad, Kjemtrup, Carreno, Thomford, Wainwright, Eberhard, Quick, Telfrom & Herwalt, 2006 while more moderately related to *Babesia conradae* Kjemtrup, Wainwright, Miller, Penzhorn & Carreno, 2006 which infects dogs. The piroplasm forms detected included tetrads (Maltese cross), merozoite and trophozoite stages whose average size was larger than stages of other canine *Babesia* spp. belonging to the *Babesia* (*s.l*.) and *B. gibsoni* Patton, 1910, and smaller than other canine *Babesia* (*sensu stricto*) spp. Of 212 *O. tholozani* ticks surveyed, 11 (5.2%) harbored DNA of the new species of *Babesia*.

**Conclusions:**

*Babesia negevi* n. sp. is described based on morphological and genetic characterization and phylogenetic analyses. The species is named after the Negev desert of southern Israel, where the first infected dog originated from. Despite co-infection in four dogs, the fifth dog had fatal disease attesting that *B. negevi* n. sp. infection requires clinical attention. Incriminating *O. tholozani* or another tick species as the vector of *Babesia negevi* n. sp., would require additional studies.
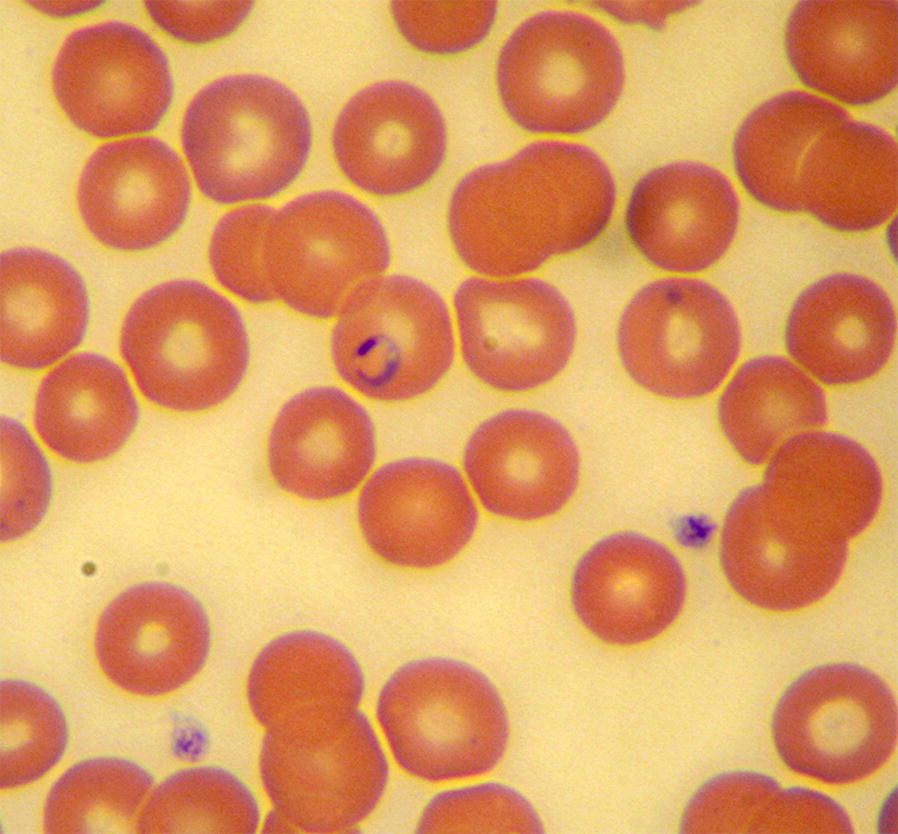

## Background

*Babesia* Starcovici, 1893 is a tick-borne protozoan genus classified in the phylum Apicomplexa, class Piroplasmea and order Piroplasmida. *Babesia* spp. infect domestic and wildlife animals, and humans, and cause severe hemolytic disease [1]. Molecular phylogeny corroborates the taxonomic entities of *Babesia* (*sensu stricto*) as a monophyletic group referred to as Clade VI while in contrast, *Babesia* (*sensu lato*) parasites represent a complex of species that can be assigned to at least two other monophyletic piroplasmid groups, designated as Clade I (“*Babesia microti*-like piroplasmids”) and Clade II (western group) [2].

Domestic dogs are infected with several *Babesia* spp. that cause severe disease and have been characterized genetically and described in detail. These include: (i) *Babesia rossi* Nuttall, 1910; (ii) *Babesia canis* Pianna & Galli-Vallerio, 1895; (iii) *Babesia vogeli* Reichenow, 1937; (iv) *Babesia gibsoni* Patton, 1910; (v) *Babesia conradae* Kjemtrup, Wainwright, Miller, Penzhorn & Carreno, 2006; and (vi) *Babesia vulpes* Baneth, Cardoso, Brilhante-Simões & Schnittger, 2019 [3–5]. The first three *Babesia* spp. present large intraerythrocytic merozoites, which are morphologically identical when examined by light microscopy while the other three species are smaller and differ in sizes and shapes among themselves. These six species vary in the severity of clinical manifestations which they cause, their tick vectors, genetic and antigenic characteristics and geographical distributions. Most of these species are known or presumed to be transmitted by hard ticks of the family Ixodidae [3–5].

*Babesia* sp. infection was detected by light microscopy of stained blood smears from five dogs in Israel. PCR of marker genes and phylogenetic analysis inferred the presence of a previously unknown species of *Babesia* in the blood of all dogs. Four of the dogs were co-infected with *Borrelia persica* (Dschunkowsky, 1913) Steinhaus, 1946, a relapsing fever agent which causes disease in humans, dogs and cats, and is transmitted by the argasid tick *Ornithodoros tholozani* Laboulbène & Mégnin [6–8]. This led to the hypothesis that this previously unknown *Babesia* sp., which was not identified in earlier molecular studies of pathogens transmitted by hard ticks in Israel and its vicinity [9, 10], might be transmitted by *O. tholozani.* We therefore surveyed soft ticks in Israel for the presence of piroplasms.

The aim of this study was to describe morphologically and characterize genetically the previously unknown piroplasmid species found to infect dogs in Israel.

## Methods

### Animal samples

Piroplasms were detected by light microscopy in blood smears stained by Romanowsky staining solutions in five dogs from Israel whose blood was tested at the Koret School of Veterinary Medicine Laboratory for vector-borne diseases in Rehovot, Israel, during 2012–2016. The medical history, physical examination findings and results of complete blood count (CBC), as well as other diagnostic and laboratory test results were extracted from the medical records of all dogs (Table 1).

**Table 1:** Demographic and clinical characteristics of dogs infected with *Babesia negevi* n. sp. included in the study

Dog number	1	2	3	4	5
Sample ID	8726; 1001	4663	9835	0408	0651; 0544
Location	Meitar	Hashmonaim	Karmei Yosef	Ashkelon	Jerusalem
Year of diagnosis	2012	2012	2013	2012	2016
Sex; age (years)	F; 3	M; 4	F; 12.5	M; 3.5	M; 0.3
Breed	Mixed breed	Labrador Retriever	Mixed breed	Labrador Retriever	Mixed breed
Fever	41.2 °C	39.5 °C	38.9 °C	39.6 °C	39.0 °C
Lethargy	+	+	+	+	+
Anorexia	+	+	+	+	−
Pale mucous membranes	+	+	+	+	+
Icterus	−	+	−	+	+
Anemia; Hematocrit; MCV (fl); MCHC (g/l)	+; 0.34 l/l; 77.7; 305	+; 0.189 l/l; 66.7; 337	+; 0.17 l/l; 69.2; 353	+: 0.13 l/l; 84.6; 288	+; 0.084 l/l; 62.3; 272
Leukocytosis; WBC (× 10^9^/l)	−; 11.80	−; 12.80	−; 9.13	+; 24.88	−; 6.45
Thrombocytopenia	+	–	+	+	+
Platelet count (× 10^9^/l)	41	171	86	4	54
Co-infection					
*Borrelia persica* infection noted on blood smear; PCR	+; +	+; +	+; +	−; −	+; +
*Ehrlichia canis* PCR	−	−	−	−	−
*Hepatozoon canis* PCR	+	−	−	−	−
Outcome	Survived	Died one day after treatment initiation	Survived	Died one day after treatment initiation	Survived
Treatment	Doxycycline and imidocarb dipropionate	Amoxicillin/ clavulanic acid; Imidocarb dipropionate	Amoxicillin; imidocarb dipropionate	Doxycycline; imidocarb dipropionate	Doxycycline; imidocarb dipropionate

*Notes*: Hematocrit, reference interval (RI), 0.371–0.57 l/l; MCV (mean corpuscular volume) RI, 58.8–71.2 fl; MCHC (mean corpuscular hemoglobin concentration) RI, 310–362 g/l; WBC (white blood cell count) RI, 5.2–13.9 × 10^9^/l; platelet count RI 143–400 × 10^9^/l

*Abbreviations*: F, female; M, male

### Collection of blood and examination of parasite morphology

Blood was collected by venipuncture of the jugular or cephalic veins of dogs into EDTA and clot tubes for hematology and serum biochemistry, respectively. Stained blood smears were examined by oil immersion light microscopy at 1000× magnification using the Nikon Eclipse N-U microscope, fitted with the Nikon DS-Ri1 camera (Nikon Corporation, Tokyo, Japan) operated by the NIS-Elements F software package (Nikon Corporation, Tokyo, Japan). Parasites were measured and photographed. All measurements are in micrometres and are given as the range followed by the mean ± standard deviation (SD) in parentheses.

### Molecular detection of *Babesia* and other tick-borne pathogens in animal samples

DNA was extracted from 200 µl of EDTA-anticoagulated blood samples from the dogs using the Illustra blood genomicPrep Mini Spin Kit (GE Healthcare, Buckinghamshire, UK), following the manufacturer’s instructions. A fragment of the piroplasmid *18S* rRNA gene was amplified by PCR using the primers PiroplasmidF (5′-CCA GCA GCC GCG GTA ATT-3′) and PiroplasmidR (5′-CTT TCG CAG TAG TTY GTC TTT AAC AAA TCT-3′) as previously described [11] and primers 522F (5′-GTT GAT CCT GCC AGT AGT-3′) and 1661R (5′-ACC TTG TTA CGA CTT CT-3′) as previously described [12] (Table 2). In addition, new primers were designed to amplify the near full length of the *18S* rRNA gene in two overlapping fragments [Fragment 1: forward primer (5′-GTT GAT CCT GCC AGT AGT-3′) and reverse primer (5′-CTG GAA AAA GAG AGC CGA-3′); Fragment 2: forward primer (5′-TGT AAT TGG AAT GAT GGG AAT C-3′) and reverse primer (5′-AAC CTT GTT ACG ACT TCT C-3′)] in order to avoid amplification of *Hepatozoon canis* DNA detected in dog no. 1. Each PCR reaction mix of 50 µl contained 1 µl of DNA template, 50 pmol of each primer, 10 nmol dNTPs, 75 nmol of MgCl_2_, 2.5 U AmpliTaq Gold DNA polymerase, and 1× GeneAmp PCR Gold Buffer (Applied Biosystems, Carlsbad, CA, USA). Thermal cycling conditions consisted of an initial denaturation at 95 °C for 5 min, followed by 50 amplification cycles (95 °C for 20 s, 62 °C for 30 s, and 72 °C for 45–90 s) and a final extension step at 72 °C for 7 min (Techne Inc., Burlington, NJ, USA). For each PCR, extension times were adjusted according to the predicted amplicon length. Amplicons were visualized on ethidium-bromide stained agarose gels, purified (QIAquick PCR Purification Kit, Qiagen Inc., Valencia, CA, USA), and sequenced bidirectionally (Genewiz, South Plainfield, NJ, USA). Additional sequencing primers (forward: 5′-TTC CGT TAA CGA ACG AGA CC-3′ and reverse: 5′-TTA TAG TTA GGA CTA CGA CGG-3′) were utilized to obtain complete bidirectional sequences for Fragment 2. Contigs were assembled using BioEdit Sequence Alignment Editor software package (North Carolina State University, Raleigh, NC, USA). Furthermore, the piroplasmid c*ox*1 gene was amplified by PCR using primers COX1F (5′-GGA AGT GGW ACW GGW TGG AC-3′) and COX1R (5′-TTC GGT ATT GCA TGC CTT G-3′) as previously described [13] (Table 2).

**Table 2:** Target genes and primers used for PCR to detect *Babesia* spp., *Borrelia persica*, *Hepatozoon canis*, *Ehrlichia canis* and *Ornithodoros tholozani* in this study

Target organism and gene	Primer	Primer sequence (5′-3′)	Reference
*Babesia 18S* rRNA	PiroplasmidF	CCAGCAGCCGCGGTAATT	[11]
	PiroplasmidR	CTTTCGCAGTAGTTYGTCTTTAACAAATCT	
	522F	GTTGATCCTGCCAGTAGT	[12]
	1661R	AACCTTGTTACGACTTCT	
	Fragment1F	GTTGATCCTGCCAGTAGT	This study
	Fragment1R	CTGGAAAAAGAGAGCCGA	
	Fragment2F	TGTAATTGGAATGATGGGAATC	
	Fragment2R	AACCTTGTTACGACTTCTC	
	AdditionalF	TTCCGTTAACGAACGAGACC	
	AdditionalR	TTATAGTTAGGACTACGACGG	
*Babesia cox*1	COX1F	GGAAGTGGWACWGGWTGGAC	[13]
	COX1R	TTCGGTATTGCATGCCTTG	
*Borrelia* spp. *flab*	Bfpbu	GCT GAA GAG CTTGGAATGCAACC	[14]
	Bfpcr	TGATCAGTTATCATTCTAATAGCA	
*Hepatozoon* spp.	Hepatozoon 18S-F	GGTAATTCTAGAGCTAATACATGAGC	[16]
	Hepatozoon 18S-R	ACAATAAAGTAAAAAACAYTTCAAAG	
*Ehrlichia* spp. *16S* rRNA	EHR16SD	GGTACCYACAGAAGAAGTCC	[15]
	EHR16SR	TAGCACTCATCGTTTACAGC	
Tick *16S* rRNA	16S+1	CTGCTCAATGATTTTTTAAATTGCTGTGG	[17]
	16S−1	CCGGTCTGAACTCAGATCAAGT	

Additionally, PCR was also performed to test the dogs for co-infection with relapsing fever *Borrelia* spp. [14], *Ehrlichia* spp. [15] and *Hepatozoon* spp. [16] (Table 2). DNA from dogs infected with *B. vogeli*, *B. persica*, *E. canis* and *H. canis* were used as positive control for the respective PCRs. DNA from a laboratory-bred dog PCR negative for *Babesia*, *Borrelia*, *Ehrlichia* and *Hepatozoon* spp. was used as a negative control and a non-template negative control (NTC) was also included in each PCR run. Positive DNA amplicons were purified (EXO-Sap, New England Biolabs Inc., Ipswich, MA, USA) and sequenced in the Center for Genomic Analyses at the Hebrew University (Jerusalem, Israel) using the BigDye Terminator cycle from Applied Biosystems ABI3700 DNA Analyzer. The ABI Data Collection and Sequence Analysis software (ABI, Carlsbad, CA, USA) was used for analysis. DNA sequences were compared to other sequences deposited on GenBank using the BLASTn website hosted by NCBI, National Institutes of Health, USA (http://www.ncbi.nlm.nih.gov) and new DNA sequences from *Babesia-*infected dogs were deposited in the GenBank database.

### Soft ticks

Argasid ticks were trapped in four caves in Israel (Beit Guvrin, Lavi, Canada Park and Nitzana) where *O. tholozani* ticks had been previously detected. Carbon dioxide traps were used to collect *O. tholozani* ticks as previously described [6]. Briefly, three collector traps connected to a cool box emitting CO_2_ from dry ice were buried in the soil in each sampling site and left overnight. On the next morning, all trapped ticks were collected with tweezers, kept in vials with 70% ethanol and brought to the laboratory for analysis. All ticks were identified morphologically as *O. tholozani* and subsequently confirmed by PCR targeting a 460-bp segment of the tick mitochondrial *16S* rRNA gene followed by DNA sequencing [17] (Table 2).

DNA was extracted from ticks using a commercial kit (DNeasy Blood & Tissue Kit, Qiagen, Hilden, Germany) following the manufacturerʼs protocol. PCR to detect the presence of *Babesia* spp. DNA in ticks was done using the piroplasmid PCR and primers (Table 2). All positive DNA amplicons were sequenced as described above and identified using BLASTn.

### Phylogenetic analyses

Phylogenetic trees were constructed based on marker gene sequences determined in this study and corresponding relevant sequences of other *Babesia* spp. deposited previously in the GenBank database. Following nucleotide sequence alignment using MUSCLE, maximum likelihood (ML) and neighbor-joining trees were inferred using MEGA version X [18]. Percentages of replicate trees as determined by 1000 bootstrap replicates (bs) are shown next to branches. A bs ≥ 85 was considered to provide strong support. Additional details of alignment and tree construction are described herein for the two ML phylograms created. The first tree was composed of 41 nearly complete *18S* rRNA gene sequences of analyzed and relevant piroplasmid species, including *Cardiosporidium cionae 18S* rRNA sequence as the outgroup. After creation of an alignment of 1592 bp in length, a ML tree was constructed based on the TN93+G+I model as estimated by using Aikaikeʼs information criterion (AIC). Based on a discrete gamma distribution consisting of five categories, the shape parameter (G = 0.49) was determined allowing for the existence of invariant sites (I = 50.5%), and the complete deletion option resulted in 1282 positions in the final dataset [19].

A piroplasmid *cox*1 tree which included 23 partial *cox*1 gene sequences of the present and other relevant piroplasmid species, with *Plasmodium falciparum cox*1 sequence as the outgroup was also inferred. After estimation of the GTR+G+I model using AIC, a discrete shape parameter was estimated based on five categories (G = 1.40) allowing for the existence of invariant sites (I = 31.6%), and the complete deletion option resulted in 463 positions in the final dataset [20].

Pairwise distance matrices were generated with multiple global alignment (Needleman-Wunsch algorithm) using the Geneiuos software, version 7.1.9 (Biomatters Ltd., Auckland, New Zealand). Results were calculated as percent identity (p-distance: identical pairs of bases/total number of pairs) [21].

## Results

**Family Babesiidae Poche, 1913**

**Genus** ***Babesia*** **Starcovici, 1893**

***Babesia negevi*** **n. sp.**

***Type-host:*** Domestic dog *Canis lupus familiaris* L. (Mammalia: Canidae).

***Type-locality:*** Town of Meitar (31°19′38.15″N, 34°56′18.78″E), Israel.

***Other localities:*** Towns of Hashmonaim (31°55′51.65″N, 35°1′17.66″E) and Karmei Yosef (31°50′53.87″N, 34°55′13.44″E) and cities of Ashkelon (31°40′N, 34°34′E) and Jerusalem (31°47′N, 35°13′E), Israel.

**Fig. 1:**
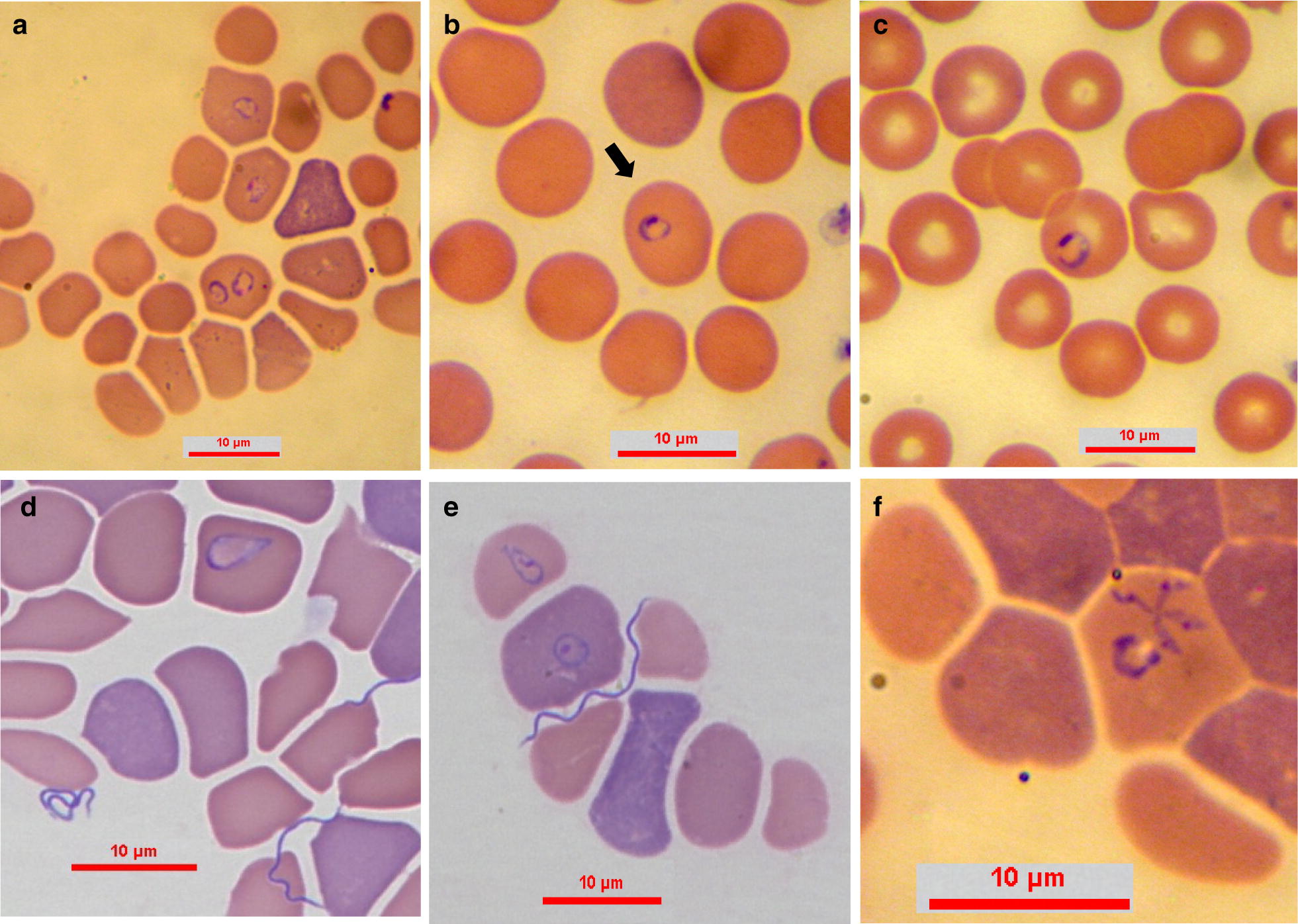
*Babesia negevi* n. sp. type-material in stained blood smears from a dog **a**, **b**. **a** Multiple *B. negevi* n. sp. parasites in the blood of infected dog. **b** Merozoite (the holotype is marked with an arrow). **c** Merozoite. **d** Trophozoite with sharp anterior end and rounded posterior end, note *B. persica* spirochetes in smear. **e** Merozoite and trophozoite in adjacent host erythrocytes, see *B. persica* spirochete between erythrocytes. **f** Tetrad and merozoite forms in the same host erythrocyte. Modified Wright’s and quick Romanowsky staining. *Scale-bars*: 10 μm

***Type-material:*** A stained thin blood smear from a 3-year-old Israeli female mixed breed dog containing the holotype (Fig. 1) was deposited in the National Natural History Collection of the Hebrew University of Jerusalem, Israel, under the accession number HUJPROTOZ1002. In addition, genomic DNA extracted from the blood of infected dogs no. 1 and 5 was deposited at the Koret School of Veterinary Medicine, Hebrew University of Jerusalem, Rehovot, Israel under the accession numbers 1001 and 0651, respectively.

***Vector:*** Unknown. The argasid tick *Ornithodoros tholozani* Laboulbène & Mégnin is suspected.

***Representative DNA sequences:*** GenBank: MN864544-MN864546 (*18S* rRNA gene); MN876837-MN876839 (*cox*1).

***ZooBank registration:*** To comply with the regulations set out in article 8.5 of the amended 2012 version of the *International Code of Zoological Nomenclature* (ICZN) [22], details of the new species have been submitted to ZooBank. The Life Science Identifier (LSID) of the article is urn:lsid:zoobank.org:pub:56DA53B2-7C55-45D9-8320-2526C2E23E52. The LSID for the new name *Babesia negevi* is urn:lsid:zoobank.org:act:D8872ABB-27EE-4C1C-989A-0F671795F8FD.

***Etymology:*** The species is named after the Negev desert of southern Israel where the first dog infected with this parasite originated from.

### Description

***Merozoites*** [Measurements based on 76 parasites; see Fig. 1a–c.] Round to oval ring-shaped merozoites with eccentric nuclei presenting as single or two intraerythrocytic parasites. Merozoites in different stages of development measuring 1.2–4.8 (2.66 ± 0.79) in length and 0.94–3.8 (2.03 ± 0.57) in width.

**Fig. 2:**
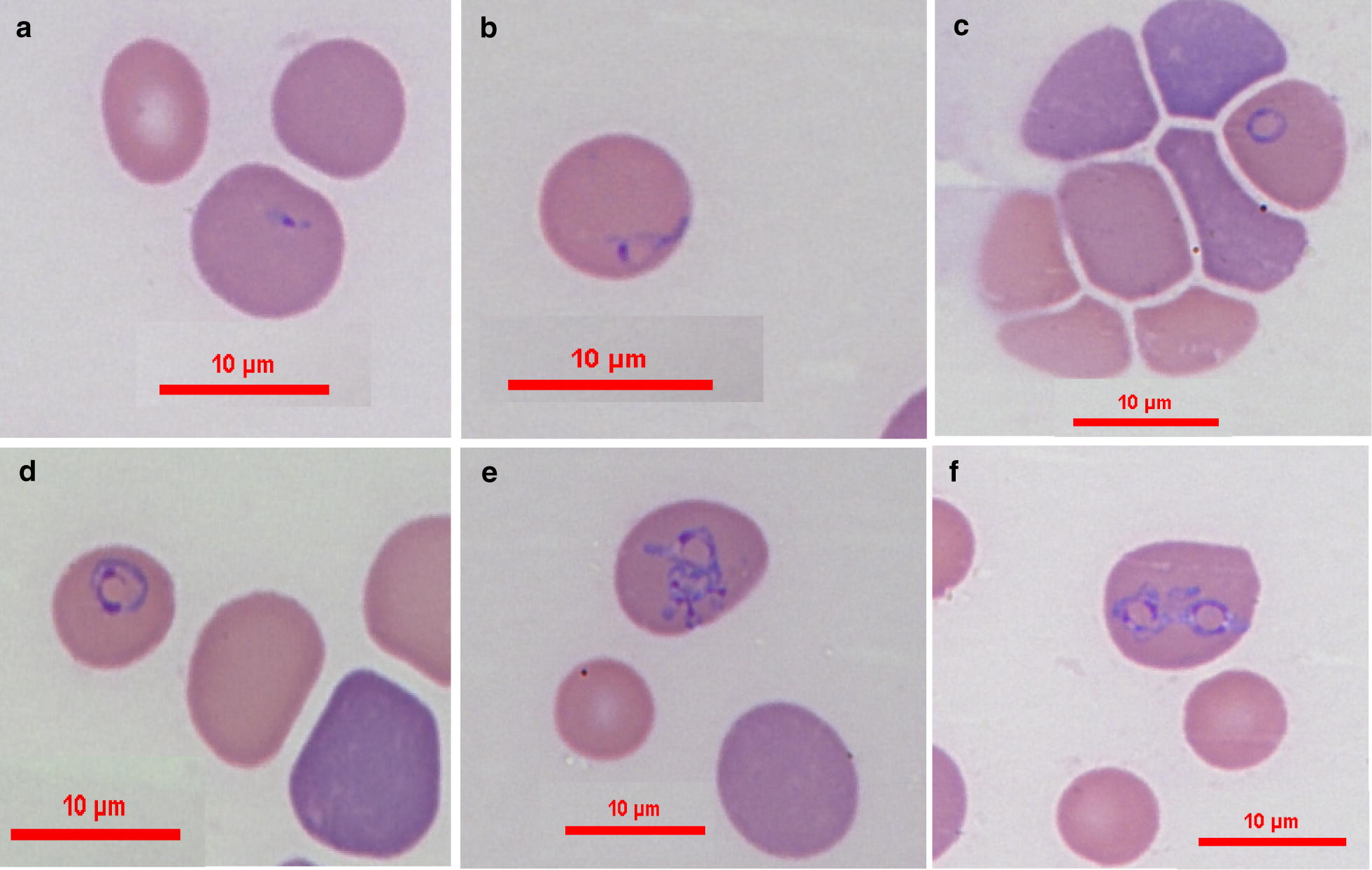
Proposed evolution of *B. negevi* n. sp. merozoite development and division. **a** Slender zoite possibly arising from a disintegrated tetrad after invading a new host cell. **b** Developing elongated trophozoite. **c** Early merozoite. **d** Merozoite. **e** Dividing merozoite. **f** Merozoites following completion of division. Blood smears stained by modified Wright’s staining solution. *Scale-bars*: 10 μm

***Trophozoites*** [Measurements based on 28 parasites; see Figs. 1d, e, 2b] Elongated forms with pointed anterior end and rounded posterior end and prominent nuclei presenting as single intraerythrocytic parasites. Trophozoites presented in different stages of development measuring 2.5–8.3 (4.46 ± 1.79) in length and 1.5–5.0 (2.34 ± 0.94) in width.

**Fig. 3:**
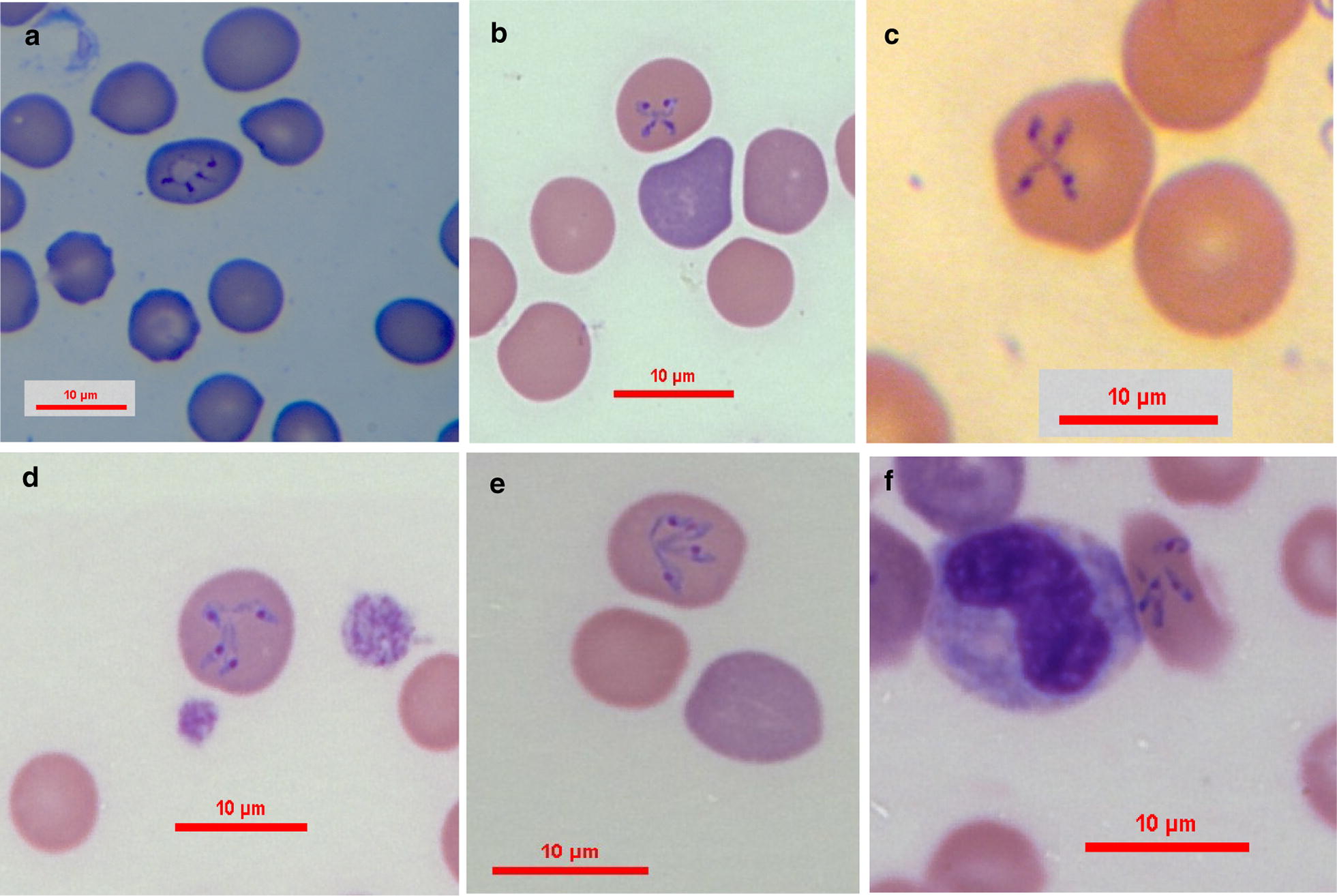
Proposed evolution of tetrad (Maltese cross) formation and disintegration. **a** A round shape central formation with four attached dark staining projections evolving to become the arms of the tetrad. **b** Tetrad with initial formation of individual nuclei. **c** Fully developed tetrad with prominent nuclei. **d**, **e** Deforming mature tetrads approaching disintegration and the detachment of four individual parasites. **f** Erythrocyte containing four individual slender parasites presumably resulting from disintegration of a tetrad. Modified Wright’s and quick Romanowsky staining. *Scale-bars*: 10 μm

***Tetrads (Maltese crosses)*** [Measurements based on 11 tetrads; see Fig. 3b, c.] Tetrads, found in the blood of all five infected dogs, included four slender parasites each with prominent round nucleus interconnected at one end to each other forming a cross shape. The tetrads measure 3.3–6.9 (4.71 ± 1.18) in length and 2.4–6.8 (3.8 ± 1.44) in width (*n* = 11). Each individual interconnected parasite measures 1.6–3.4 (2.34 ± 0.65) in length and 0.4–0.3 in width (*n* = 44).

### Differential diagnosis

Intraerythrocytic parasites presented in several shapes. Merozoites of *B. negevi* n. sp. are round to oval ring-shaped and appear to be encircled by a basophilic-staining layer of variable width around a less densely stained pale center (Fig. 1b, c). This peripheral basophilic staining layer has single or multiple wider and denser staining chromatin nuclei with larger more mature merozoites often having more than a single dense chromatin nucleus. Mature merozoites divide by binary fission forming pairs of merozoites in the same erythrocyte (Fig. 2f). The presumptive development of mature merozoites and their division is outlined in Figs. 1 and 2. Small slender zoites such as those arising from disintegrated tetrads invade the erythrocyte (Fig. 2a) and enlarge to develop into early trophozoites (Fig. 2b) which develop further into larger trophozoites with a pointed posterior end (Fig. 1d, e). Trophozoites become round to oval ring-shaped merozoites (Fig. 2c, d) and eventually begin to divide by binary fission (Fig. 2e) until they form two individual rounded merozoites within the erythrocyte (Fig. 2f). The presumptive development of tetrads is shown in Fig. 3. An early form develops from a rounded parasite which develops four dark-staining projections (Fig. 3a). The rounded form shrinks, leaving four interconnected zoites with prominent nuclei (Fig. 3b) which elongate to form a full Maltese cross tetrad shape (Fig. 3c). The zoites may deform and bend (Fig. 3d, e) until they dissociate from each other (Fig. 3f) and are ready to break free from their host erythrocyte and infect additional erythrocytes (Fig. 2a).

The forms of *B. negevi* n. sp. described here from dog erythrocytes are morphologically similar to the forms described for *B. conradae* described from dogs in California [23], and also bear resemblance to other small canine-infecting *Babesia* spp. such as *B. gibsoni* [24] and *B. vulpes* [5]. Nevertheless, the merozoites of *B. negevi* n. sp. measuring on average 2.66 × 2.03 µm are larger than the ring forms described for *B. conradae* which measure 2.2 × 1.85 µm, and also larger than the pyriform shapes of *B. conradae* which measure 1.38 × 0.66 µm [23]. They are also larger than the merozoites of *B. vulpes* which measure on average 1.33 × 0.98 µm [5] and the forms of *B. gibsoni*, measuring 1.9 × 1.2 µm [25]. Conversely, the merozoites of *B. negevi* n. sp. are distinctly smaller than the merozoites of canine *Babesia* spp. producing large merozoites including *Babesia vogeli*, *Babesia canis* and *Babesia rossi*, which typically measure 4.5–5.0 × 2.0–2.5 µm. The tetrad form of *B. negevi* n. sp. measured 4.71 × 3.8 µm which is larger than that reported for *B. conradae* (2.5 × 2.0 µm) [23]. Importantly, tetrad forms such as those found in *B. negevi* sp. nov, were observed only in *B. conradae* [23] and in none of the other species of *Babesia* spp. infecting dogs.

The above comparisons indicate that *B. negevi* n. sp. is a distinct form consistent with the small-form piroplasms infecting canines. *Babesia negevi* n. sp. forms tend to be larger than their respective life stages in *B. conradae*, *B. vulpes* and *B. gibsoni*, and smaller than the large-form *Babesia* spp. infecting canines including *B. canis*, *B. vogeli* and *B. rossi*.

### Molecular phylogeny

The nearly complete piroplasmid *18S* rRNA gene (about 1700 bp) was amplified from the blood of dogs no. 1, 2 and 4 (GenBank: MN864546, MN864544 and MN864545, respectively), while a somewhat shorter sequence of 962 bp was amplified from dog no. 3 (GenBank: MN864547). Pairwise comparisons showed a 99–100% identity (Additional file 1: Table S1). All five dogs also yielded shorter *18S* rDNA sequences of about 330 bp with the piroplasmid primers PCR protocol (Table 2) which showed 100% identity with respect to each other (GenBank: MN864539-MN864543). As determined by BLASTn, the closest matches of 94–97% identity to all *Babesia* spp. *18S* rDNA sequences isolated from the dogs in this study included a sequence of a *Babesia* sp. from a wild meerkat (*Suricata suricatta*) in South Africa (GenBank: KM025199), and a similarly high identity of 97% was found between sequences for *B. negevi* n. sp. from dogs no. 1, 3, and 4, and a sequence of *B. duncani* Conrad, Kjemtrup, Carreno, Thomford, Wainwright, Eberhard, Quick, Telfrom & Herwalt, 2006 from a human in the USA (GenBank: HQ289870.1) (Additional file 1: Table S1).

**Fig. 4:**
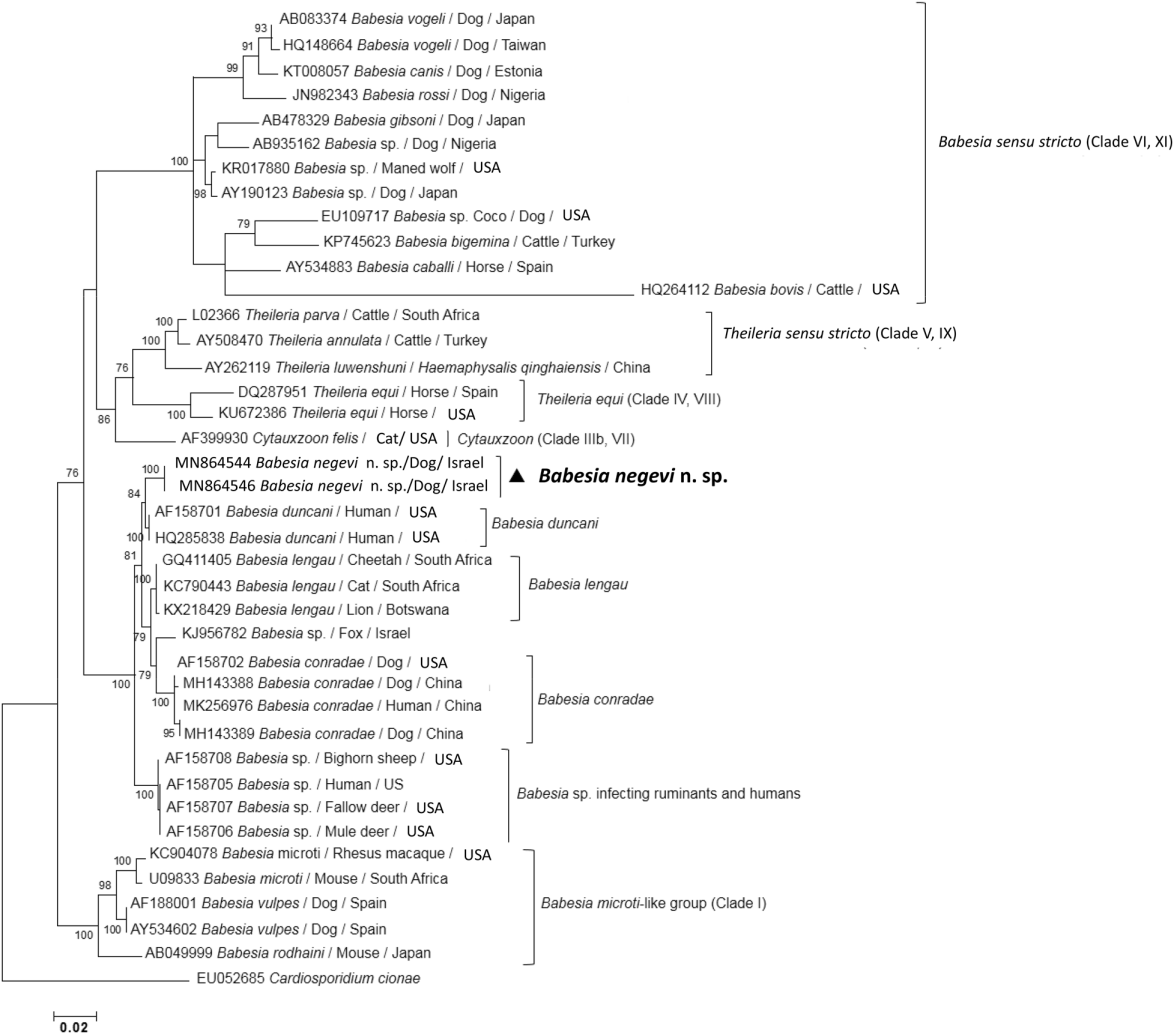
A maximum likelihood phylogenetic tree of nearly complete *18S* rRNA gene sequences. The first piroplasmid clade designation is given as defined previously in Schnittger et al. (2012) [28, 31] whereas the second Roman number corresponds to a recently revised novel clade designation [2]. The GenBank accession number, host and country of origin are included for each sequence. Only bootstrap values > 70 are indicated next to branches. The scale-bar represents the evolutionary distance in the units of the number of nucleotide substitutions per site

A phylogenetic tree based on nearly complete *18S* rRNA gene sequences was inferred using the ML algorithm and including *B. negevi* n. sp. and other piroplasmids present on GenBank representing a wide range of parasites of the order Piroplasmida, including all those that have been found to infect dogs (Fig. 4). Sequences of *B. negevi* n. sp. were recovered within the western group of *Babesia* spp. into a distinct, strongly supported sister clade (bs: 100) sister to *B. duncani* (bs: 84) [26]. Furthermore, the *B. negevi* n. sp. clade is placed more distantly from the sequences representing the *B. conradae* clade (bs: 100) and from the clade (bs: 100) represented by *B. lengau* Bosman, Oosthuizen, Peirce, Venter & Penzhorn, 2010 [27]. Finally, within the western group, a *Babesia* sp. that has been reported from ruminants and humans (GenBank: AF158705-AF158708), represents a strongly supported sister clade (bs: 100) to all other species and is thus most distantly related to *B. negevi* n. sp. (Clade II, III) [2, 28]. Other more distant piroplasmid lineages, some of which contain dog-infecting piroplasmids, include *Babesia* (*s.s*.) (Clade VI; XI), *Babesia microti*-like group (Clade I), *Cytauxzoon* spp. (Clade IIIb, VII), *Theileria* (*s.s*.) (Clade V, IX), and *Theileria equi* (Clade IV, VIII) [2, 28]. A neighbor-joining phylogram of the same sequences analyzed by ML showed corresponding topology and bootstraps to the ML analysis (Additional file 2: Figure S1).

*18S* rDNA *Babesia* sequences were amplified from 13 of 212 (6.1%) studied *O. tholozani* ticks and submitted to GenBank (GenBank: MN864547-MN864569). Of these, 11 (5.2%) were compatible with *B. negevi* n. sp., while the remaining two were different, with one sequence (GenBank: MN864559) closely related (96% identity) to a sequence of a *Babesia* sp. amplified from a dog in Angola (GenBank: KX082919) and a second sequence (GenBank: MN864560) closely related (99% identity) to a *Babesia* sp. from a badger in China (GenBank: MG799846) (Additional file 1: Table S1). All 16 sequences of the hypervariable region of the *18S* rRNA gene of the five dog-derived sequences of *B. negevi* n. sp. (GenBank: MN864539-MN864543) and the eleven tick-derived sequences (GenBank: MN864548-MN864558) showed a 99–100% identity to each other by pairwise comparison thus identifying them as *B. negevi* n. sp. (Additional file 3: Table S2). An additional pairwise comparison of a 332 bp sequence of the hypervariable *18S* rRNA gene region of *B. negevi* n. sp., with other closely related *Babesia* spp. including all known to infect dogs showed that *B. negevi* n. sp. had a genetic distance of at least 5.42% from the most closely related species *B. duncani* (Additional file 4: Table S3).

**Fig. 5:**
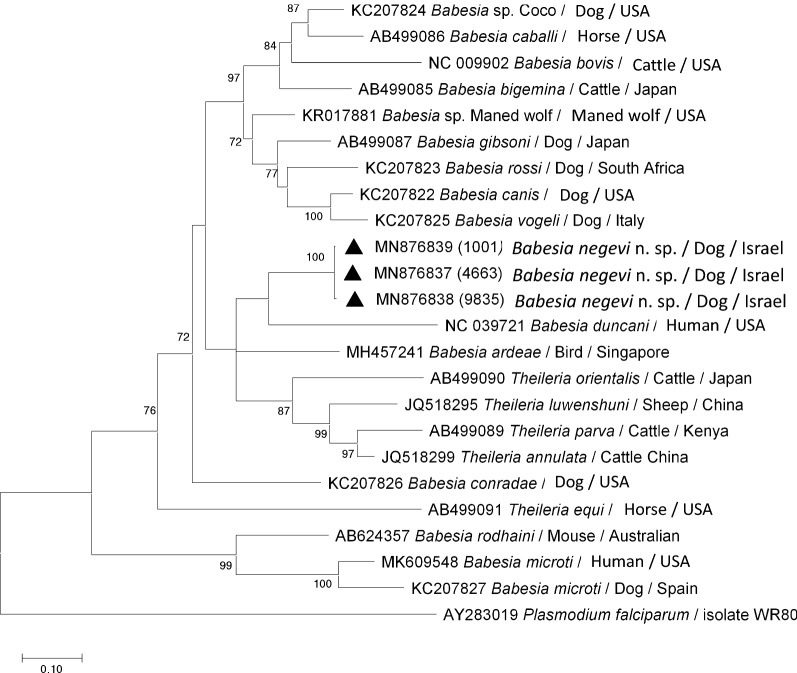
A maximum likelihood phylogram based on 463-bp-long *cox*1 gene fragments of *Babesia* spp. isolates from dogs (black triangle) of this study including other relevant piroplasmid species deposited in GenBank. The GenBank accession number, host and country of origin are included for each sequence. Only bootstrap values > 70% are indicated. The scale-bar represents the evolutionary distance in the units of the number of nucleotide substitutions per site

Mitochondrial *cox*1 gene sequences of 463, 727 and 905 bp were amplified from *B. negevi* n. sp.-infected dogs no. 1, 3, and 2, respectively (GenBank: MN876839, MN876838 and MN876837, respectively; Additional file 1: Table S1), and showed an identity of 99–100% to each other. A phylogenetic tree of 463-bp long mitochondrial *cox*1 gene alignment (Fig. 5) of the three *cox*1 sequences of *B. negevi* n. sp. amplified from dogs with corresponding relevant *cox*1 nucleotide sequences of other piroplasmids resulted in a strongly supported clade (bs: 100) corroborating the identity of *B. negevi* n. sp. as a novel species. However, due to short marker sequences resulting in a reduced phylogenetic signal, the placement of *B. negevi* n. sp. as a sister clade to *B. duncani* was not strongly supported (bs < 70). Of other piroplasmid lineages, *Theileria equi* was moderately supported (bs: 76), whereas *Theileria* (*s.s*.) (bs: 87), *Babesia* (*s.s*.) (bs: 97), and the *B. microti*-like group (bs: 99) displayed a strong support.

Recently, *Babesia behnkei* Bajer, Alsarraf, Bednarska, Mohallal, Mierzejewska, Behnke-Borowczyk, Zalat, Gilbert & Welc-Falęciak, 2014 has been described from Wagenr’s gerbils (*Dipodilus dasyurus*) from the Sinai Peninsula of Egypt, which is situated close to Israel [29]. The *18S* rDNA sequence of this species exhibits an identity of only 95% with that of *B. negevi* n. sp. No *cox*1 sequence of this species was available for comparison.

Overall, the *18S* rRNA gene phylogeny constructed using nearly complete gene sequences clearly demonstrated that *B. negevi* n. sp. represents a novel distinct species that is distinguished from other known piroplasmid species available on GenBank and particularly from those infecting canines. *Babesia negevi* n. sp. is placed within the western group as a sister clade with respect to *B. duncani*. Importantly, pairwise comparison of the *18S* rRNA gene hypervariable region of *B. negevi* n. sp. from dogs and *O. tholozani* ticks demonstrated the identity of these isolates. In addition, the phylogenetic tree based on *cox*1 sequences of *B. negevi* n. sp. amplified from infected dogs further corroborated the identity and distinct status of this species.

Interestingly, a study on hemoparasites of dogs from the Palestinian Authority, adjacent to Israel, has reported three dogs infected by unidentified *Babesia* spp. In a phylogenetic analysis of partial *18S* rRNA gene sequences, their corresponding sequences segregated jointly with *B. conradae* into two additional distinct well supported clades within the western group of *Babesia* spp. [30]. Piroplasmid sequences amplified in the Palestinian study from two dogs and designated 33.5 and 24 clustered into a sister branch to that of *B. conradae*, whereas an additional piroplasmid sequence from the third dog designated 30.1 represented a more distantly related sister species to *B. conradae* and piroplasmid spp. 33.5 and 24. Although the piroplasmid sequences originating from these dogs have not been deposited in the GenBank database, we were able to receive these sequences by personal communication (Additional file 5: Table S4). A pairwise comparison of *B. negevi* n. sp. to the Palestinian dog piroplasmid sequences indicated that sequences 33.5 and 24 displayed a high identity of 99.7% with *B. negevi* n. sp. whereas sequence 30.1 showed a substantially lower identity of only 92.5% (Additional file 5: Table S4). This finding indicates that sequences 33.5 and 24 most probably represent *B. negevi* n. sp.

### Clinical findings in infected dogs and tick survey

The infected dogs included three males and two females ranging in age from three months to twelve years, including two Labrador Retrievers and three mixed breed dogs (Table 1). On clinical examination, abnormalities included lethargy and pale mucous membranes (5/5 dogs), fever (temperature > 39.0 °C; 4/5 dogs), decreased appetite (4/5 dogs), and icterus (3/5 dogs). On CBC all dogs were anemic, two with overt regenerative macrocytic hypochromic anemia (dogs no. 1 and 4), two with normocytic normochromic anemia (dogs no. 2 and 3) and one with normocytic hypochromic anemia (dog no. 5). Four of the five dogs were thrombocytopenic and the fifth dog had a normal thrombocyte count which was close to the low reference interval. The leukocyte count was within the reference interval in four of the five dogs, while moderate leukocytosis was noted in one. Co-infection with *B. persica* was evident on blood smear (Fig. 1d, e) and confirmed by PCR and DNA sequencing in four of the five dogs, while dog no. 4 was negative for borreliosis by both diagnostic techniques. All dogs were negative by PCR for *E. canis*, which is a common cause of anemia and thrombocytopenia in Israel, and dog no. 1 was also co-infected with *H. canis* as detected by PCR and DNA sequencing. All dogs were treated with the antiprotozoal imidocarb dipropionate and with an antibiotic against relapsing fever borreliosis, either doxycycline (dogs no. 1 and 4) or amoxicillin/clavulanic acid (dogs no. 2 and 5) or just amoxicillin (dog no. 3). Three dogs (no. 1, 3 and 5), all co-infected with *B. persica*, survived the infection while two dogs, no. 2 co-infected with *B. persica* and no. 4 infected only with *B. negevi* n. sp. died one day after the beginning of treatment.

A total of 212 *O. tholozani* ticks were collected in four caves: Nitzana (*n *= 38) in southern Israel, Beit Guvrin (*n *= 69) and Canada Park (*n *= 55) in central Israel, and Lavi in northern Israel (*n *= 50). Seven *O. tholozani* positive for *B. negevi* n. sp. by PCR were trapped in the Beit Guvrin cave and four in Lavi cave (*n *= 11; 5.1%). Of the remaining two *Babesia* sp. sequences different from *B. negevi* n. sp. detected in ticks, one was from Beit Guvrin and the other from Lavi.

## Discussion

This study describes *B. negevi* n. sp. as a new taxon fulfilling the requirements of the ICZN guidelines for a new species [22]. The placement of *B. negevi* n. sp. in the genus *Babesia* and its segregation in the western clade of *Babesia* spp. within this genus is derived from the molecular phylogenetic analysis of the *18S* rRNA and *cox*1 gene sequences. These phylogenetic findings clearly demonstrate the distinct species status of *B. negevi* n. sp. as corroborated by the demonstration of a single strongly supported clade in each of the constructed phylogenetic trees. The nearly identical *18S* rRNA gene fragment sequences amplified from *O. tholozani* ticks and dogs from different locations indicate that *B. negevi* n. sp. is spread geographically in several areas in Israel. The description of two dogs from the Palestinian study [30] infected with previously unidentified *Babesia* sp. which upon analysis performed in this study showed a similar identity of 99.7% to *B. negevi* n. sp. further supports the information on the spread and geographical distribution of this species. The placement of *B. negevi* n. sp. as a sister clade of *B. duncani* within the western group of *Babesia* as demonstrated by the nearly complete *18S* rRNA gene tree provides an interesting insight on the global spread of the western group babesiae. *Babesia duncani*, a species that infects humans in western North America [26], appears to be the closest known relative of *B. negevi* n. sp. by both *18S* rRNA and *cox*1 phylogenetic analyses. *Babesia conradae*, which infects dogs in California, is another western group species found to be somewhat less closely related to *B. negevi* n. sp. [23]. *Babesia duncani* and *B. conradae* segregate into moderately related independent clades which are both strongly supported (bs: 100). Importantly, both of these species have been originally identified in the western part of the North American continent. Thus, these two species have not yet been reported in Israel nor in any other geographical region of the Middle East. As the understanding of the different clades within the Piroplasmida is evolving with more research on the genetics and life-cycles of different parasites [2, 13, 28, 31], it is possible that the current genus *Babesia* may be split in the future to reflect clade differences.

*Babesia negevi* n. sp., *B. conradae* and *B. duncani* all produce tetrads. This distinguishes *B. negevi* n. sp. and *B. conradae* from the other described *Babesia* spp. that infect dogs. Nevertheless, the average size of *B. conradae*’s tetrads is smaller than the average size described for the tetrads of *B. negevi* n. sp., which is also in agreement with the size differences of other comparable life stages of these two species such as merozoites.

The vector of *B. negevi* n. sp. is currently unknown and the detection of its DNA in *O. tholozani* in the present study is not sufficient to prove that this tick species is its vector. However, the fact that four of five dogs with *B. negevi* n. sp. infection were co-infected with *B. persica*, for which *O. tholozani* is a vector [6], and that previous molecular surveys of *Babesia* spp. in hard ticks in Israel and the adjacent Palestinian Authority have not reported the presence of DNA compatible with *B. negevi* n. sp. strengthen the idea that *O. tholozani* could be its vector [9, 10].

Although ixodid ticks are the known vectors of *Babesia* spp. [1], the bat soft tick *Argas vespertilionis* is a suspected vector of *Babesia vesperuginis* [32], the only piroplasm currently known to infect bats, which is genetically closely related to *B. conradae* [33]. Furthermore, *Ornithodoros moubata* soft ticks infected by *B. gibsoni* by injection of the parasite into the tick’s hemocoel have been shown to transmit *B. gibsoni* to dogs after attaching to their skin and feeding on them [34]. An earlier attempt to infect *O. moubata* by feeding through a parafilm membrane on horse blood infected with *T. equi* in that same study failed, presumably because the parasite did not penetrate the tick’s gut to disseminate further. Based on these two experiments, it has been suggested that some piroplasmid parasites transmitted in nature *via* hard ticks may be transferred also by soft ticks, if they succeed to escape the tick’s gut defense mechanisms and the midgut barrier [34]. Nevertheless, the possibility of natural transmission of *B. negevi* n. sp. by *O. tholozani* should be further tested by experimental methods to confirm or reject this hypothesis.

Babesiosis in dogs is frequently a severe and life-threatening disease [3]. Understanding the outcomes of *B. negevi* n. sp. infection in the five dogs described in this study is complicated by the fact that four of them were co-infected with *B. persica*, which causes disease in humans, cats and dogs, associated with fever, lethargy, anemia and occasional thrombocytopenia [6–8]. Although all five dogs in this study suffered from a severe disease, dog no. 4 who was negative for *B. persica*, had a typical acute babesiosis presenting a remarkable parasitemia, fever, severe regenerative hemolytic anemia and thrombocytopenia, and died despite antiprotozoal and supportive treatment. These findings attest to the potential involvement of *B. negevi* n. sp. with overwhelming pathological consequences. Therefore, despite the confounding co-infection found in most of the reported dogs, *B. negevi* n. sp. should be regarded a canine pathogen requiring clinical attention, and once detected, there should be a search for co-infection with *B. persica* as a possible accompanying pathogen.

## Conclusions

This study describes a new *Babesia* species infecting dogs in the Middle East, which is part of the western *Babesia* species group and is associated with severe clinical disease. More research is warranted to reveal the vectors of *B. negevi* n. sp., other potential animal hosts and the most effective treatment and prevention of infection with this disease agent.

## Supplementary information

**Additional file 1: Table S1**. GenBank accession numbers of all DNA sequences produced in the study detailing samples number, host, gene, total length in base pairs, closest GenBank match, nucleotide identities, % identity and % coverage.

**Additional file 2: Figure S1.** A neighbor joining phylogenetic tree based on nearly complete *18S* rRNA gene sequences. The first piroplasmid clade designation is given as defined previously in Schnittger et al. (2012) [28, 31] whereas the second Roman number corresponds to a recently revised novel clade designation [2]. The GenBank accession numbers, host and country of origin are included for each sequence.

**Additional file 3: Table S2.** Pairwise distance matrix of 233 bp of the hypervariable *18S* rRNA gene region amplified from dogs and ticks in this study was conducted using the multiple global alignment option in the Geneiuos software, version 7.1.9 [21]. The GenBank accession numbers of all sequences are included, dog numbers and their sample number are in parentheses and tick sequences are designated as OT (*Ornithodors tholozani*) and the tick’s specific number. The sequences obtained from 11 ticks and 5 dogs had 99–100% identity to each other.

**Additional file 4: Table S3.** Pairwise distance matrix comparing 332 bp of *18S* rDNA sequence of *Babesia negevi* n. sp. (MN864539) to other *Babesia* spp. was conducted using the multiple global alignment option in the Geneiuos software, version7.1.9 [21]. Data represent % identity (p-distance).

**Additional file 5: Table S4.** Partial *18S* rRNA gene sequences obtained from the blood of Palestinian dogs included in a phylogram in Azmi et al. (2016) [30], kindly provided by Dr Kifaya Azmi. A pairwise nucleotide comparison between the hypervariable *18S* rRNA gene region of *Babesia negevi* n. sp. with corresponding sequences originating from three *Babesia* sp. isolates obtained from the Palestinian Authority is included [29].

## Data Availability

All data generated or analyzed during this study are included in this published article. Analyzed nucleotide sequences used for pairwise comparisons and tree construction were submitted to the GenBank database under the accession numbers MN864539-MN864560 (*18S* rRNA gene) and MN876837-MN876839 (*cox*1). The holotype was deposited in the National Natural History Collection of the Hebrew University of Jerusalem, Israel, under the accession number HUJPROTOZ1002.

## Abbreviations

bs: bootstrap
*cox*1: cytochrome *c* oxidase subunit 1 gene
ICZN: International Code of Zoological Nomenclature
PCR: polymerase chain reaction
*s.l.*: *sensu lato*
*s.s*.: *sensu stricto*
