# Supplementary material for: A new piroplasmid species infecting dogs: morphological and molecular characterization and pathogeny of Babesia negevi n. sp
Source: Parasit Vectors. 2020 Apr 21;13:130. doi: 10.1186/s13071-020-3995-5 (PMC7171826; doi:10.1186/s13071-020-3995-5)
Supplement: Supplementary file 1 — Additional file 1: Table S1. GenBank accession numbers of all DNA sequences produced in the study detailing samples number, host, gene, total length in base pairs, closest GenBank match, nucleotide identities, % identity and % coverage. [file 13071_2020_3995_MOESM1_ESM.docx]

| Accession number | Sample^a^ | Host | Gene | Total length (bp) | Closest Genbank match^b^ | Nucleotide Identities (bp) | % identity | % Cover |  |
| --- | --- | --- | --- | --- | --- | --- | --- | --- | --- |
| MN864539 | 0408 | Dog 4 | 18S | 332 | KM025199.1 Uncultured *Babesia* (Suricata, South Africa) | 314/331 | 95% | 99% |  |
| MN864540 | 9835 | Dog 3 | 18S | 330 | KM025199.1 Uncultured *Babesia* (Suricata, South Africa) | 312/330 | 95% | 99% |  |
| MN864541 | 1001 | Dog 1 | 18S | 329 | KM025199.1 Uncultured *Babesia* (Suricata, South Africa) | 311/328 | 95% | 99% |  |
| MN864542 | 0544 | Dog 5 | 18S | 328 | KM025199.1 Uncultured *Babesia* (Suricata, South Africa) | 308/327 | 94% | 99% |  |
| MN864543 | 4663 | Dog 2 | 18S | 418 | KM025199.1 Uncultured *Babesia* (Suricata, South Africa) | 399/418 | 95% | 100% |  |
| MN864544 | **4663** | Dog 2 | 18S | 1698 | KM025199.1 Uncultured *Babesia* (Suricata, South Africa) | 1631/1677 | 97% | 98% |  |
|  |  |  |  |  | HQ289870.1 *Babesia duncani* (Human, USA) | 1652/1706 | 97% | 100% |  |
| MN864545 | 0408 | Dog 4 | 18S | 1698 | KM025199.1 Uncultured *Babesia* (Suricata, South Africa) | 1631/1677 | 97% | 98% |  |
|  |  |  |  |  | HQ289870.1 *Babesia duncani* (Human, USA) | 1652/1706 | 97% | 100% |  |
| MN864546 | **1001** | Dog 1 | 18S | 1635 | AF158701.1 Piroplasmida gen. sp. (Human, USA) | 1593/1644 | 97% | 100% |  |
|  |  |  |  |  | HQ289870.1 *Babesia duncani* (Human, USA) | 1592/1643 | 97% | 100% |  |
| MN864547 | 9835 | Dog 3 | 18S | 1029 | KM025199.1 Uncultured *Babesia* (Suricata, South Africa( | 974/1035 | 96% | 98% |  |
| MN864548 | OT_310n | *O. tholozani* | 18S | 233 | KJ956779.3 *Babesia* sp. MML (Golden jackal, Israel) | 222/235 | 94% | 100% |  |
| MN864549 | OT_243n | *O. tholozani* | 18S | 233 | KJ956779.3 *Babesia* sp. MML (Golden jackal, Israel) | 222/235 | 94% | 100% |  |
| MN864550 | OT_324n | *O. tholozani* | 18S | 233 | KJ956779.3 *Babesia* sp. MML (Golden jackal, Israel) | 223/235 | 95% | 100% |  |
| MN864551 | OT_343n | *O. tholozani* | 18S | 234 | KJ956779.3 *Babesia* sp. MML (Golden jackal, Israel) | 222/236 | 94% | 100% | |
| MN864552 | OT_227f | *O. tholozani* | 18S | 233 | KJ956779.3 *Babesia* sp. MML (Golden jackal, Israel) | 222/235 | 94% | 100% | |
| MN864553 | OT_162n | *O. tholozani* | 18S | 233 | KJ956779.3 *Babesia* sp. MML (Golden jackal, Israel) | 222/235 | 94% | 100% | |
| MN864554 | OT_325n | *O. tholozani* | 18S | 233 | KJ956779.3 *Babesia* sp. MML (Golden jackal, Israel) | 222/235 | 94% | 100% | |
| MN864555 | OT_330n | *O. tholozani* | 18S | 235 | KJ956779.3 *Babesia* sp. MML (Golden jackal, Israel) | 223/238 | 94% | 100% | |
| MN864556 | OT_112n | *O. tholozani* | 18S | 233 | KJ956779.3 *Babesia* sp. MML (Golden jackal, Israel) | 223/235 | 95% | 100% | |
| MN864557 | OT_136m | *O. tholozani* | 18S | 233 | KJ956779.3 *Babesia* sp. MML (Golden jackal, Israel) | 223/235 | 95% | 100% | |
| MN864558 | OT_332f | *O. tholozani* | 18S | 233 | KJ956779.3 *Babesia* sp. MML (Golden jackal, Israel) | 223/235 | 95% | 100% | |
| MN864559 | OT_336f | *O. tholozani* | 18S | 237 | KX082919.1 *Babesia* sp. (Dog, Angola) | 223/233 | 96% | 98% |  |
| MN864560 | OT_314n | *O. tholozani* | 18S | 235 | MG799846.1 *Babesia* sp. (Badger, China) | 220/222 | 99% | 94% |  |
| MN876837 | **4663** | Dog 2 | Cox1 | 905 | KC207821.1 *Cytauxzoon felis* (Cat, USA) | 720/910 | 79% | 100% |  |
|  |  |  |  |  | KR017881.1 *Babesia* sp. (maned wolf, South America) | 682/856 | 80% | 94% |  |
| MN876838 | **9835** | Dog 3 | Cox1 | 727 | MF996533.1 *Babesia vesperuginis* (Bat, Czech Republic) | 531/649 | 82% | 88% |  |
| MN876839 | **1001** | Dog 1 | Cox1 | 463 | MK592916.1 *Babesia* sp. (Raccoon, USA) | 368/457 | 81% | 98% |  |

**Additional file 1: Table S1**. GenBank accession numbers of all DNA sequences produced in the study detailing sample number, host, gene, total length in base pairs, closest GenBank match, nucleotide identities, % identity and % coverage.

a. Sequences in bold are included in the phylogenetic trees; b. One or two first GenBank matches by BLAST are included.

All “short” (233 bp) 18S *rRNA* sequences are 99-100% identical to each other. All “long” (956 bp) 18S *rRNA* sequences are 99-100% identical to each other. All *cox*1 sequences (464bp) are 99-100% identical to each other.
