# Supplementary material for: A new piroplasmid species infecting dogs: morphological and molecular characterization and pathogeny of Babesia negevi n. sp
Source: Parasit Vectors. 2020 Apr 21;13:130. doi: 10.1186/s13071-020-3995-5 (PMC7171826; doi:10.1186/s13071-020-3995-5)
Supplement: Supplementary file 2 — Additional file 2: Figure S1. A neighbor joining phylogenetic tree based on nearly complete 18S rRNA gene sequences. The first piroplasmid clade designation is given as defined previously in Schnittger et al. (2012) [28, 31] whereas the second Roman number corresponds to a recently revised novel clade designation [2]. The GenBank accession numbers, host and country of origin are included for each sequence. [file 13071_2020_3995_MOESM2_ESM.pptx]

## Slide 1
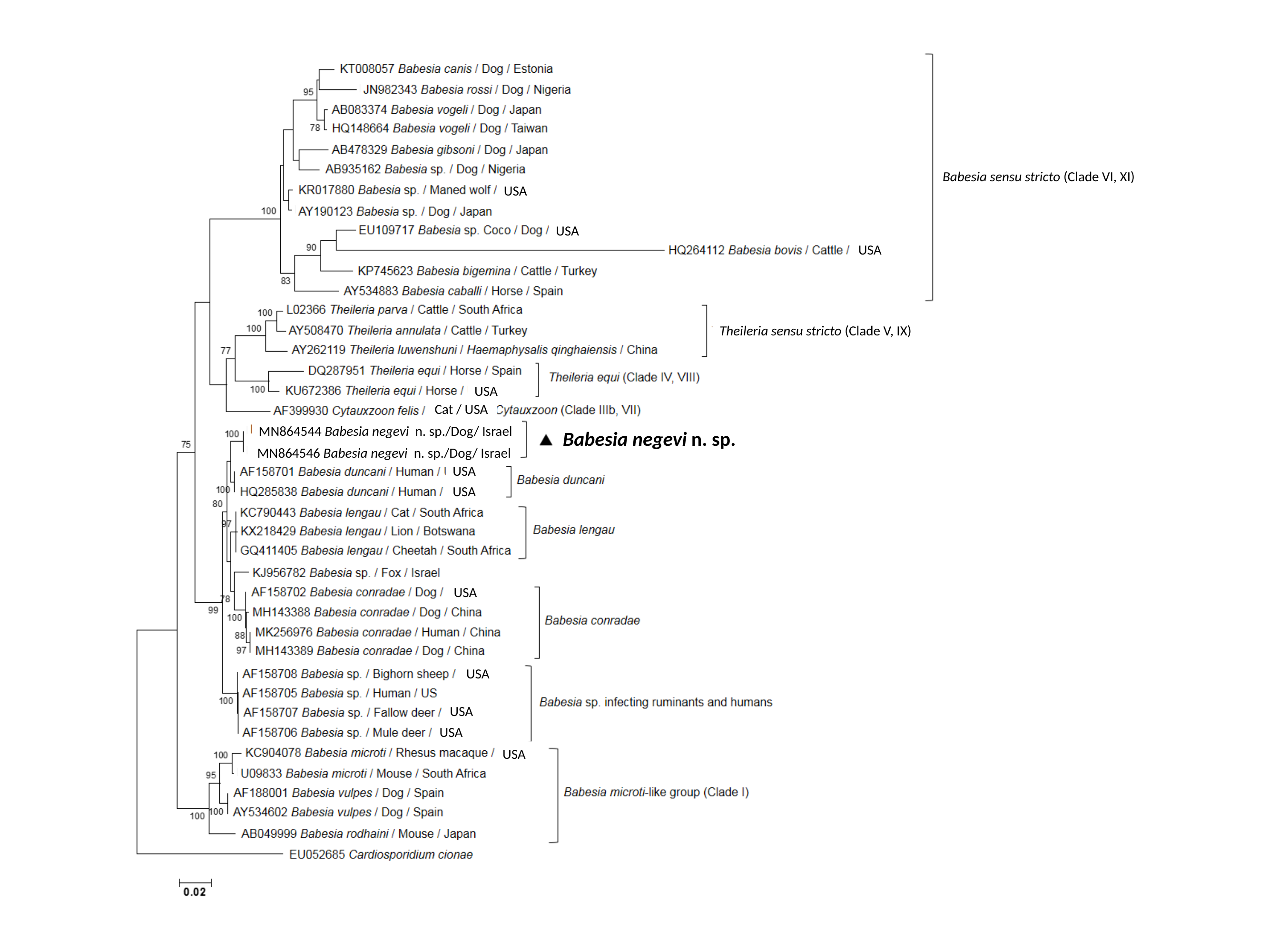

Babesia negevi n. sp. / Dog / Israel
 Babesia negevi n. sp.
Babesia negevi n. sp. / Dog / Israel
Babesia sensu stricto (Clade VI, XI)
USA
USA
USA
Theileria sensu stricto (Clade V, IX)
USA
Cat / USA
USA
USA
USA
USA
USA
USA
USA
MN864544 Babesia negevi n. sp./Dog/ Israel
MN864546 Babesia negevi n. sp./Dog/ Israel
USA

## Slide 2
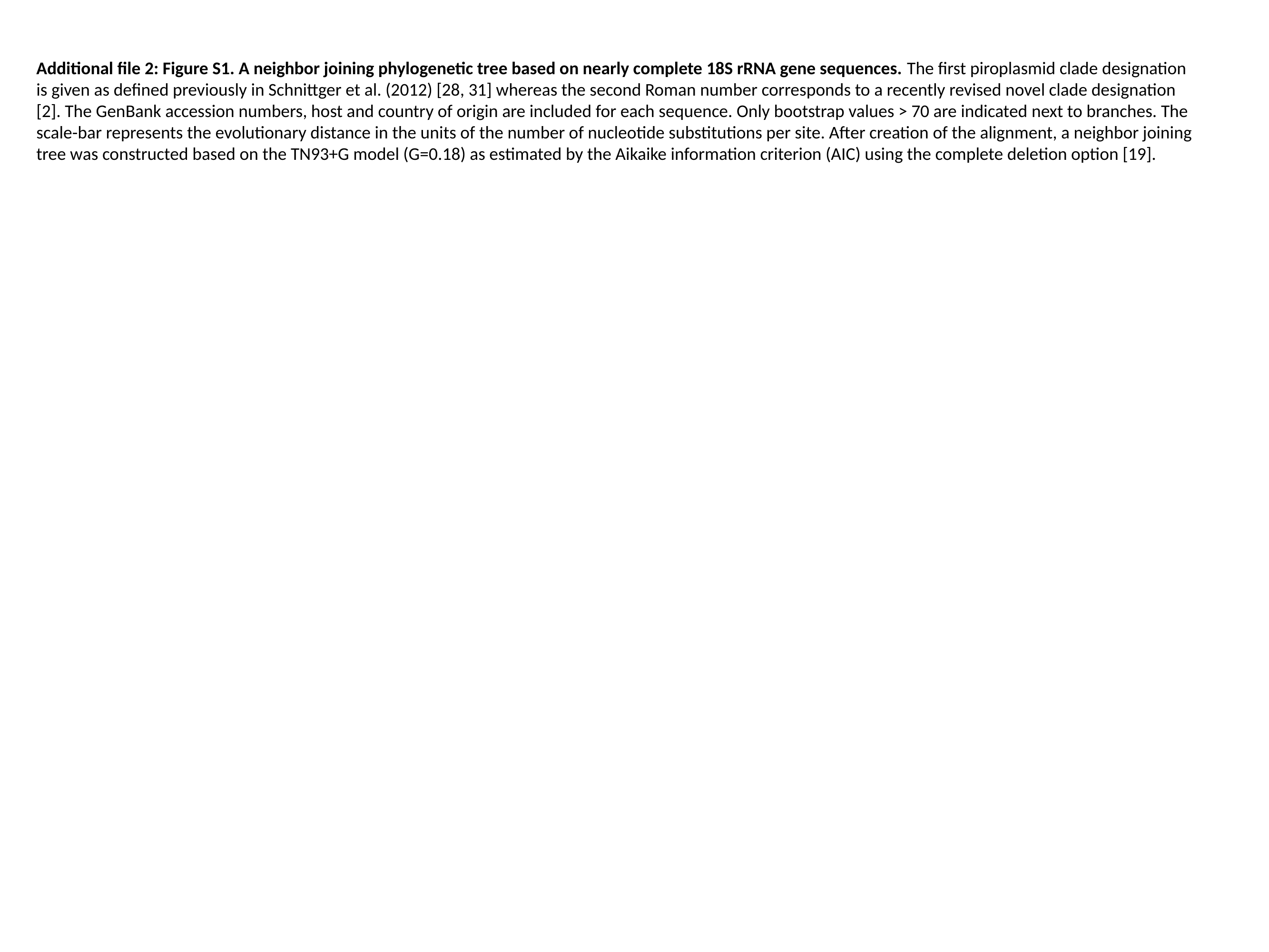

Additional file 2: Figure S1. A neighbor joining phylogenetic tree based on nearly complete 18S rRNA gene sequences. The first piroplasmid clade designation is given as defined previously in Schnittger et al. (2012) [28, 31] whereas the second Roman number corresponds to a recently revised novel clade designation [2]. The GenBank accession numbers, host and country of origin are included for each sequence. Only bootstrap values > 70 are indicated next to branches. The scale-bar represents the evolutionary distance in the units of the number of nucleotide substitutions per site. After creation of the alignment, a neighbor joining tree was constructed based on the TN93+G model (G=0.18) as estimated by the Aikaike information criterion (AIC) using the complete deletion option [19].
