# Supplementary material for: A new piroplasmid species infecting dogs: morphological and molecular characterization and pathogeny of Babesia negevi n. sp
Source: Parasit Vectors. 2020 Apr 21;13:130. doi: 10.1186/s13071-020-3995-5 (PMC7171826; doi:10.1186/s13071-020-3995-5)
Supplement: Supplementary file 3 — Additional file 3: Table S2. Pairwise distance matrix of 233 bp of the hypervariable 18S rRNA gene region amplified from dogs and ticks in this study was conducted using the multiple global alignment option in the Geneiuos software, version 7.1.9 [21]. The GenBank accession numbers of all sequences are included, dog numbers and their sample number are in parentheses and tick sequences are designated as OT (Ornithodors tholozani) and the tick’s specific number. The sequences obtained from 11 ticks and 5 dogs had 99–100% identity to each other. [file 13071_2020_3995_MOESM3_ESM.docx]

**Additional file 3: Table S2.** Pairwise distance matrix of 233 bp of the hypervariable 18S *rRNA* gene region amplified from dogs and ticks in this study was conducted using the multiple global alignment option in the Geneiuos software, version 7.1.9 (Biomatters Ltd., Auckland, New Zealand) [21]. Data are given as % identity. The GenBank accession numbers of all sequences are included, dog numbers and their sample number are in parentheses and tick sequences are designated as OT (*Ornithodors tholozani*) and the tick’s specific number. The sequences obtained from 11 ticks and 5 dogs had 99-100% identity to each other.

|  | MN864543 (dog 2, 4663) | MN864539 (dog 4, 0408) | MN864540 (dog 3, 9835) | MN864541 (dog 1, 1001) | MN864542 (dog 5, 0544) | MN864550 (OT 324n) | MN864555 (OT 330n) | MN864554 (OT 325n) | MN864549 (OT 243n) | MN864548 (OT 310n) | MN864552 (OT 227f) | MN864553 (OT 162n) | MN864551 (OT 343n) | MN864557 (OT 136m) | MN864556 (OT 112n) | MN864558 (OT 332f) | MN864559 (OT 336f) |
| --- | --- | --- | --- | --- | --- | --- | --- | --- | --- | --- | --- | --- | --- | --- | --- | --- | --- |
| MN864539 (dog 4, 0408) | 100 |  |  |  |  |  |  |  |  |  |  |  |  |  |  |  |  |
| MN864540 (dog 3, 9835) | 100 | 100 |  |  |  |  |  |  |  |  |  |  |  |  |  |  |  |
| MN864541 (dog 1, 1001) | 100 | 100 | 100 |  |  |  |  |  |  |  |  |  |  |  |  |  |  |
| MN864542 (dog 5, 0544) | 100 | 100 | 100 | 100 |  |  |  |  |  |  |  |  |  |  |  |  |  |
| MN864550 (OT 324n) | 100 | 100 | 100 | 100 | 100 |  |  |  |  |  |  |  |  |  |  |  |  |
| MN864555 (OT 330n) | 99.11 | 99.11 | 99.11 | 99.11 | 99.11 | 99.11 |  |  |  |  |  |  |  |  |  |  |  |
| MN864554 (OT 325n) | 99.55 | 99.55 | 99.55 | 99.55 | 99.55 | 99.55 | 99.56 |  |  |  |  |  |  |  |  |  |  |
| MN864549 (OT 243n) | 99.55 | 99.55 | 99.55 | 99.55 | 99.55 | 99.55 | 99.56 | 100 |  |  |  |  |  |  |  |  |  |
| MN864548 (OT 310n) | 99.55 | 99.55 | 99.55 | 99.55 | 99.55 | 99.55 | 99.56 | 100 | 100 |  |  |  |  |  |  |  |  |
| MN864552 (OT 227f) | 99.55 | 99.55 | 99.55 | 99.55 | 99.55 | 99.55 | 99.56 | 100 | 100 | 100 |  |  |  |  |  |  |  |
| MN864553 (OT 162n) | 99.55 | 99.55 | 99.55 | 99.55 | 99.55 | 99.55 | 99.56 | 100 | 100 | 100 | 100 |  |  |  |  |  |  |
| MN864551 (OT 343n) | 99.11 | 99.11 | 99.11 | 99.11 | 99.11 | 99.11 | 99.12 | 99.56 | 99.56 | 99.56 | 99.56 | 99.56 |  |  |  |  |  |
| MN864557 (OT 136m) | 100 | 100 | 100 | 100 | 100 | 100 | 99.11 | 99.55 | 99.55 | 99.55 | 99.55 | 99.55 | 99.11 |  |  |  |  |
| MN864556 (OT 112n) | 100 | 100 | 100 | 100 | 100 | 100 | 99.11 | 99.55 | 99.55 | 99.55 | 99.55 | 99.55 | 99.11 | 100 |  |  |  |
| MN864558 (OT 332f) | 100 | 100 | 100 | 100 | 100 | 100 | 99.11 | 99.55 | 99.55 | 99.55 | 99.55 | 99.55 | 99.11 | 100 | 100 |  |  |
| MN864559 (OT 336f) | 87.61 | 87.61 | 87.61 | 87.61 | 87.61 | 87.61 | 88.5 | 88.05 | 88.05 | 88.05 | 88.05 | 88.05 | 87.67 | 87.61 | 87.61 | 87.61 |  |
| MN864560 (OT 314n) | 70.13 | 70.13 | 70.13 | 70.13 | 70.13 | 70.13 | 71 | 70.56 | 70.56 | 70.56 | 70.56 | 70.56 | 70.26 | 70.13 | 70.13 | 70.13 | 68.53 |
