# Supplementary material for: A new piroplasmid species infecting dogs: morphological and molecular characterization and pathogeny of Babesia negevi n. sp
Source: Parasit Vectors. 2020 Apr 21;13:130. doi: 10.1186/s13071-020-3995-5 (PMC7171826; doi:10.1186/s13071-020-3995-5)
Supplement: Supplementary file 4 — Additional file 4: Table S3. Pairwise distance matrix comparing 332 bp of 18S rDNA sequence of Babesia negevi n. sp. (MN864539) to other Babesia spp. was conducted using the multiple global alignment option in the Geneiuos software, version7.1.9 [21]. Data represent % identity (p-distance). [file 13071_2020_3995_MOESM4_ESM.docx]

**Additional file 4: Table S3.** Pairwise distance matrix comparing 332 bp of *18S* rDNA sequence of *Babesia negevi* n. sp. (MN864539) to other *Babesia* spp. was conducted using the multiple global alignment option in the Geneiuos software, version7.1.9 [21]. Data represent % identity (p-distance).

| Species | Accession number | Pairwise distance | |
| --- | --- | --- | --- |
|  |  | % identity | no. identities (bp) |
| *Babesia conradae* | MK256976 | 94.58 | 314 |
| *Babesia duncani* | KX008042 | 90.96 | 302 |
| *Babesia vesperuginis* | MK934426 | 89.52 | 297 |
| *Babesia vulpes* | MK957183 | 81.42 | 270 |
| *Babesia gibsoni* | MH620356 | 79.70 | 265 |
| *Babesia vogeli* | KX082917 | 79.10 | 263 |
| *Babesia canis* | MN134074 | 78.57 | 261 |
| *Babesia rossi* | MH143395 | 80.48 | 267 |
