# Supplementary material for: A new piroplasmid species infecting dogs: morphological and molecular characterization and pathogeny of Babesia negevi n. sp
Source: Parasit Vectors. 2020 Apr 21;13:130. doi: 10.1186/s13071-020-3995-5 (PMC7171826; doi:10.1186/s13071-020-3995-5)
Supplement: Supplementary file 5 — Additional file 5: Table S4. Partial 18S rRNA gene sequences obtained from the blood of Palestinian dogs included in a phylogram in Azmi et al. (2016) [30], kindly provided by Dr Kifaya Azmi. A pairwise nucleotide comparison between the hypervariable 18S rRNA gene region of Babesia negevi n. sp. with corresponding sequences originating from three Babesia sp. isolates obtained from the Palestinian Authority is included [29]. [file 13071_2020_3995_MOESM5_ESM.docx]

**Additional file 5: Table S4.** Partial *18S* rRNA gene sequences obtained from the blood of Palestinian dogs included in a phylogram in Azmi et al. (2016) [30], kindly provided by Dr Kifaya Azmi. A pairwise nucleotide comparison between the hypervariable *18S* rRNA gene region of *Babesia negevi* n. sp. with corresponding sequences originating from three *Babesia* sp. isolates obtained from the Palestinian Authority is included [29]. A 332 bp fragment of the *18S* rRNA gene sequence of *Babesia negevi* n. sp. (MN864539) was compared to the corresponding fragments of *Babesia* spp. sequences 24, 33.5, and 30.1 isolated from three dogs from the Palestinian Authority. Multiple sequence alignment of *18S* rRNA gene sequences of corresponding length was done and pairwise genetic distances estimated employing the p-distance model using the [MegaX](file:///C:\Users\yaari\Downloads\MegaX) software [18]. Data represent % identity (p-distance) between sequences.

| Dog sample number | Partial 18S *rRNA* gene sequence |
| --- | --- |
| 30.1 | GTTTATGGTTAGGACTACGACGGTATCTGATCGTCTTCGAGCCCCTAACTTTCGTTCTTGATTAATGAAAACATCCTTGGCAAATGCTTTCGCAGTAGTTCGTCTTTAACAAATCTAAGAATTTCACCTCTGACAGTTAAATACGAATGCCCCCAACTGTTCCTATTAACCATTACTTTGGTCCTGAAACCAACAAAATAGAACCAAAGTCCTACTTTATTATTCCATGCTGAAGTATTCAAGGCAAAAGCCTGCTTGAAACACTCTAATTTTCTCAAAGTAAAGCTGGAAAAAGAGAGCCGAAGTATCAAATTAACCGGAGGAAGGCCAGCCAGCAAACGTGGCCACAGGGAGAACCAAGTCCGCTGTCCTTTAGTTCATGTACGAGCTTTTTAACTGCAACAAGTTTAATATACGCTATTGGAGCTGGAATTACCGCGGCTGC-GGCACCAGAC |
| 24 | GTTTATGGTTAGGACTACGACGGTATCTGATCGTCTTCGAGCCCCTAACTTTCGTTCTTGATTAATGAAAACATCCTTGGCAAATGCTTTCGCAGTAGTTCGTCTTTAACAAATCTAAGAATTTCACCTCTGACAGTTAAATACGAATGCCCCCAACTGTTCCTATTAACCATTACTTTGGTCCTGAAACCAACAAAATAGAACCAAAGTCCTACTTTATTATTCCATGCTGAAGTATTCAAGGCAAAAGCCTGCTTGAAACACTCTAATTTTCTCAAAGTAAAGCTGGAAAAAGAGAGCCGAAGCTCAAATTAACCAGAGGTATGCCACCAGGATAATGTAGCCCCAAGGGGAACAAAGTCCTGGCGGCAGAAGTTCAACTACGAGCTTTTTAACTGCAACAAGTTTAATATACGCTATTGGAGCTGGAATTACCGCGGCTGCTGGCACCAGAC |
| 33.5 | GTTTATGGTTAGGACTACGACGGTATCTGATCGTCTTCGAGCCCCTAACTTTCGTTCTTGATTAATGAAAACATCCTTGGCAAATGCTTTCGCAGTAGTTCGTCTTTAACAAATCTAAGAATTTCACCTCTGACAGTTAAATACGAATGCCCCCAACTGTTCCTATTAACCATTACTTTGGTCCTGAAACCAACAAAATAGAACCAAAGTCCTACTTTATTATTCCATGCTGAAGTATTCAAGGCAAAAGCCTGCTTGAAACACTCTAATTTTCTCAAAGTAAAGCTGGAAAAAGAGAGCCGAAGCTCAAATTAACCAGAGGTATGCCACCAGGATAATGTAGCCCCAAGGGGAACAAAGTCCTGGCGGCAGAAGTTCAACTACGAGCTTTTTAACTGCAACAAGTTTAATATACGCTATTGGAGCTGGAATTACCGCGGCTGCGGCACCAGAC |

|  | *Babesia negevi* n. sp. | 24 | 33.5 |
| --- | --- | --- | --- |
| 24 | 99.7 |  |  |
| 33.5 | 99.7 | 100 |  |
| 30.1 | 92.5 | 92.8 | 92.8 |
